# The Importance of Learning with Patients: Post-Pandemic Takeaways on Learning Professionalism in Clinical Settings

**DOI:** 10.21315/mjms2024.31.1.12

**Published:** 2024-02-28

**Authors:** Rita Mustika, Anyta Pinasthika, Nadia Greviana

**Affiliations:** Medical Education Collaboration Cluster, Indonesia Medical Education, and Research Institute (IMERI), Faculty of Medicine, Universitas Indonesia, Indonesia

**Keywords:** professionalism, medical education, clinical clerkship, medical student, curriculum

## Abstract

**Background:**

Public demands for high-quality healthcare require medical schools to ensure that physicians attain various competencies, including professionalism and humanism. This can be accomplished through various interactions and socialisations within the healthcare community. These meaningful learning experiences become more critical as students face unpredictable learning opportunities in clinical settings. However, professional development focuses on lapses, remediation and knowledge retention rather than its practice. To nurture professional and humanistic physicians, this study explores how medical students perceive learning professionalism in clinical settings.

**Methods:**

This is a qualitative phenomenology study involving medical students in clinical rotations at the Faculty of Medicine Universitas Indonesia. Respondents were chosen through a purposive sampling method that considered their gender and clinical years. Data were collected through focus group discussions (FGDs) and thematic analysis was used.

**Results:**

Three FGDs were conducted with 31 clinical students. Learning professionalism in clinical settings presents challenges, including the hidden curriculum (HC), limited exposure to patients and the clinical learning environment because of the social restrictions caused by the COVID-19 pandemic. The tailored strategy to learn professionalism in the clinical learning environment was more teacher-driven, including role modelling, debriefing, providing feedback and teaching context-specific knowledge on professionalism, followed by patient interactions.

**Conclusion:**

The significance of students’ interactions with the clinical learning environment, especially with patients and clinical teachers as role models, is the key to learning professionalism in clinical settings. This finding is an important takeaway in curriculum design for professionalism.

## Introduction

Considering the complexity of healthcare systems, the increasing demands for high-quality healthcare require the complete readiness of medical doctors who have sufficient knowledge, skills and attitudes for medical practice. Therefore, medical schools must ensure the attainment of these competencies, which require dynamic adaptation in educating and training future fit-for-practice medical doctors and lifelong learners (1).

Basic principles of professionalism, including excellence, accountability, altruism, humanism, ethics, integrity and awareness of healthcare systems, are essential and should be nurtured through meaningful learning processes during pre-clinical and clinical years (2–5). Nurturing humanistic values during the study of medicine is key to developing professionalism (5, 6). The nurturing process can be done through a wide range of interactions with teachers, peers, colleagues and the healthcare community in which students immerse themselves through socialisation processes, including mentorship and reflective practice (7, 8).

These meaningful learning experiences become more critical as students face unpredictable learning opportunities in clinical settings. As learning in the clinical setting is conducted in a workplace-based, service-oriented manner that directly involves students providing patient care within a limited amount of time, students may be confronted with dilemmas that affect their professional development (9, 10). The lack of relevance to clinical practice has been a challenge in developing undergraduate medical students’ professionalism (11). In addition, professional development programmes in medical school curricula have focused more on professionalism lapses, remediation (12, 13), and knowledge retention of the concept of professionalism rather than its practice (14). Therefore, arguably, teaching humanism and professionalism should be conducted explicitly during clinical years when professional values may be compromised (14).

A study conducted in the hierarchical culture context showed that students agreed that the curriculum is an important aspect of professional identity formation. It was further highlighted that the explicit teaching and assessment of professionalism, a supportive and nurturing learning environment and workplace-based learning—to some extent—affected students’ professional development (15). As medical students are the main stakeholders in medical education, they play essential roles in developing medical school curricula, including the appraisal of the existing curriculum related to professional development during the clinical stage. By sharing their experiences and perspectives on learning, students have identified situational challenges and suggested solutions and innovations for the curriculum (16, 17). Therefore, this study aims to explore how medical students perceive learning professionalism in clinical settings.

## Methods

### Context

Faculty of Medicine Universitas Indonesia (FMUI) undergraduate medical doctor programme consists of a seven-semester academic phase and a four-semester clinical phase. Students complete four long clinical rotations (internal medicine, surgery, child and adolescent health and maternal health) for about a year before completing eight shorter clinical rotations during another year. Upon completion of the 12 clinical rotations, students undergo a 6-month pre-internship clinical placement (PICP) in primary healthcare settings. Professionalism and humanism are taught longitudinally in the academic and clinical phases. During each year of the academic phase, students complete Empathy, Ethics and Professionalism modules that teach concepts and knowledge of professionalism. In the clinical phase, teaching professionalism is integrated within each clinical rotation through workplace-based learning and discussions, and its assessment is embedded in workplace-based assessment. A portfolio assessment at the end of all clinical rotations was also conducted.

### Study Design and Respondents

This is a qualitative phenomenology study involving Year 5 and Year 6 medical students at FMUI to explore their perceptions of learning professionalism in clinical settings. This study was conducted from August 2022 to December 2022. Respondents were chosen through the maximum variety sampling method, which considered gender, number of past clinical rotations and the clinical year. We purposely selected students in one minor clinical rotation so that respondents had finished at least four long rotations and students in the pre-internship clinical placement.

Students undergoing the minor clinical rotation were enrolled from different years. Therefore, past clinical rotations varied among respondents. Some were in their final rotation (the last two rotations) among the short rotation programme, others were at the beginning (within the first three rotations) of the short rotation programme, while some were in the fourth to sixth short rotation. Hence, we ensured the exploration of a variety of learning experiences of humanism and professionalism in different clinical rotations, settings and venues. Meanwhile, students from the pre-internship clinical placement were included as respondents to explore insights into professional dilemmas in the primary healthcare settings in which they are going to serve society after graduating as medical doctors.

We received the names of students enrolled in the respective clinical rotation and pre-internship clinical placement from clinical rotation administrators and invited them to join the focus group discussions (FGDs) through the electronic chat platform. All students participating in this study signed electronic consent forms.

### Data Collection and Analysis

Three FGDs were conducted online for about 1 h to 1.5 h for each FGD in Bahasa Indonesia. All FGDs were video-recorded through Zoom meeting features and moderated by the second and third authors (AP and NG)—medical educationalists are not involved in any teaching learning or assessment of clinical students. Table 1 lists the questions used during the FGDs and Table 2 lists the participants. Recordings were transcribed verbatim and analysed through the thematic analysis approach using the SCAT matrix of Otani’s steps for coding and the theorisation model on the Microsoft Excel page. The subthemes were identified by determining noteworthy phrases mentioned by respondents (18). Relevant identified subthemes were then grouped into themes through iterative discussions involving all authors.

## Results

### Respondents’ Characteristics

Three FGDs were conducted with 31 clinical students with the following demographic traits: 22 Year 5 and 9 Year 6 clinical students; 19 females and 12 males. Respondents’ characteristics can be viewed in Table 2.

### Main Themes

This study identified three main themes regarding: i) challenges to learning professionalism, ii) a tailored strategy for learning professionalism befitting the clinical learning environment and iii) making humanism and professionalism explicit in the curriculum.

### Challenges to Learning Professionalism

Learning professionalism in clinical settings presents some challenges and this study identified two main ones: i) the hidden curriculum (HC) and ii) online learning because of the COVID-19 pandemic.

#### Hidden Curriculum

The HC theme has subthemes focused on learning, the workload of students and residents, learning culture, and negative role models. Professionalism and humanism were not cornerstones of clinical learning for students or teachers, as learning still focused on achieving competence in clinical skills.

“It cannot be denied that professionalism is not the number one priority in learning here [in the clinical rotation] … I see that some students do not see professionalism as a concern … more concern is put on memorising pathophysiology or clinical reasoning” (F3).

The workload also presented a challenge to learning professionalism in clinical settings. Heavy workloads prevented students from acting professionally. This also applied to residents who were role models of professionalism for the students.

“Maybe one of the challenges [in being professional] is the workload that makes us tired and [we] cannot really talk to the patient in a better way. The same goes for residents” (F2).

Different learning cultures in various clinical departments affected how students learned professionalism. This may be related to how clinical teachers act as role models.

“How they [teachers] respond to patients … really shapes your attitude … because every single person is different and every [learning] environment has different cultures. Like, some departments or divisions interact a lot with patients, but some do not … because they’re dealing directly with the patient’s therapy [procedural skills] … Those little differences sound trivial, but [they have] an impact” (F1).

Negative role models were a profound subtheme of the HC theme. They were essential to learning professionalism and caused further confusion and dilemmas for students.

“At that time, I was confused about what we should do. There was an experience when … the clinical teacher yelled at the patient until they [the patient] cried, but we did not know what to do” (F3).

“If there were bad [examples], it’s also okay because we know there are bad and good [examples], so we know from two sides. If we don’t know [that] one is bad, we would not know that one is good” (F2).

#### Online Learning due to the COVID-19 Pandemic

The COVID-19 pandemic and subsequent social restrictions also affected how students learned professionalism. The implementation of online and blended learning methods restricted patient interactions and the clinical learning environment. The students had limited opportunities and time to interact with patients and were exposed to a limited variety of patients. This lack of opportunities made students feel less confident and “unprofessional” in dealing with patients.

“So, we could not … stay too long [with the patient]. We had to wear lots of PPE [Personal Protective Equipment] to keep ourselves and the patient safe. We could not be too close [to the patient], and we [had] to examine them carefully” (F2).

“At that time, because our rotation in internal medicine and surgery [had] no night shifts, I still cannot feel ‘professional,’ especially in emergency settings” (F3).

### Tailored Strategy for Learning Professionalism in a Clinical Learning Environment

To overcome these challenges, this study identified strategies that can be implemented and tailored for clinical settings, including providing: i) direct patient interaction, ii) positive role models, iv) debriefing and iv) context-specific knowledge.

Direct patient interaction is one of the most critical post-pandemic takeaways for learning professionalism. Students appreciated this interaction as a way to practice and sharpen their professionalism.

“Meeting directly with patients [for learning professionalism]. That’s way more helpful than just case simulations” (F3).

“When we visit the polyclinic or the ER and see the patients [who] are being treated, we can see that these patients are the ones who are suffering from the illness that we used to study [in the classroom]” (F3).

Positive role modelling by clinical teachers was considered an irreplaceable method of teaching professionalism in clinical settings.

“I think professionalism is not taught explicitly … but from clinical teachers who asked [students] to be on time … so it’s not directly taught but applied. So, we learn from the experience” (F2).

“We’re not taught [about professionalism] explicitly, but we’re taught through observation. So, the [clinical teacher] will do it. First, we observe, and we know the how-to and how to do it right” (F2).

Debriefing was identified as a strategy for learning professionalism and was conducted by providing feedback on student professionalism after direct patient interaction in addition to feedback on clinical reasoning and procedures.

“After we meet the patient, we often discuss [their] illness with the doctor [clinical teacher] … maybe it can also be added about professionalism, [such as], ‘If you said it like that, it’s not good, you should have said it like this instead’… I think it’s feasible … it does not need that much time” (F2).

For students to learn professionalism, clinical teachers should also debrief them about unexpected clinical experiences.

“Not all things can be accepted as [they are]. If we find something [that is] counterproductive or negative, I think we should try to digest it by ourselves first. But … maybe we can discuss it like we’re doing right now” (F1).

Students mentioned that teaching professionalism in clinical settings should be supported by context-specific or discipline-based knowledge. Students found that knowledge of professionalism should be discussed using specific patient characteristics or prominent professional elements in each discipline or clinical rotation.

“To increase our professionalism, it would be better if the modules could arrange a lecture about professionalism that is specific to the [clinical rotation]. Because the problems in every clinical rotation, the [patients’ characteristics] are different, the professional dilemma is different, such as in internal medicine or in OBGYN, they have different problems regarding professionalism” (F2).

“Instead of repeating the [knowledge about] professionalism as a whole, we could give pitfalls [in professionalism] that are usually met in this clinical rotation. STDs—it’s clear that it’s sensitive information. But how about pregnant women? How would they behave? How should we behave around them?” (F3).

### Making Humanism and Professionalism Explicit in the Curriculum

This study identified some takeaway points for designing a professionalism curriculum for clinical settings. Teaching and learning professionalism in clinical settings should be explicitly mentioned in the formal curriculum to ensure that it is a priority and a focus in learning.

“About professionalism, maybe it should be included in the assessment or the topics that need to be addressed [in learning]” (F3).

“So far, we learn professionalism implicitly. It means that we have to conclude it by ourselves, ‘Oh, so what they mean by professionalism is this’” (F2).

To facilitate the learning of professionalism, there needs to be a clear standard of medical doctors’ professionalism that can be referred to by students and teachers.

“The problem is that there are no standards in modelling that apply to all [teachers]. As mentioned, we can’t generalise every single clinical teacher. So maybe that’s why my experience in learning professionalism is different from my other friends because there’s no standard in [learning professionalism]” (F2).

Curriculum design for professionalism should consider a longitudinal design that integrates teaching professionalism into every clinical rotation. This also enables the curriculum to include context-specific content, as mentioned earlier.

“I agree that the module should be longitudinally held. Maybe it would be more effective if it’s specific to every clinical rotation. So, there will be professionalism in internal medicine, in child health, and it would be easier to apply in specific clinical rotations” (F2).

In addition to the integrated curriculum design, the assessment of professionalism should be explicitly embedded in various workplace-based assessments. Direct observation and feedback have become more critical to learning professionalism.

“I think there are several modules that … in miniCEX and DOPS … where we meet the patient, the way we take history, and how we interact with the patient is being observed. I think that can also be one of the methods in learning professionalism” (F3).

In support of this curriculum, faculty development efforts should focus on having the same approach among clinical teachers in teaching professionalism and role modelling. Clinical teachers should also begin to focus on professionalism in learning.

“Maybe what’s lacking is the training [for] clinical teachers because they do not have the same way [about professionalism], so that everyone can have the same perception about what to do [in teaching professionalism]” (F3).

“I think it should be highlighted to clinical teachers to not only talk about academics but also more about professionalism. Also, about role models, we think it’s still lacking, lacking [in showing] us good professionalism … That clinical teachers have to be an example for us [students]” (F3).

Faculty development efforts should also focus on designing specific professionalism topics according to each clinical department’s expertise.

“Maybe the clinical teachers need to have some training about how to teach professionalism to the students … Clinical teachers do not need to have the same way of assessing, but which specific parts need to be highlighted on empathy and professionalism, I think” (F3).

## Discussion

Teaching professionalism in medical education is focused on supporting the professional identity formation of students, as teaching professionalism is also known as character building (5, 7). Professional identity formation engages students in the socialisation process, thereby changing their existing identities into personal and professional identities. This process is influenced by numerous factors, including role models, experiential learning, teaching professionalism, and the learning environment (7, 19). The findings of this study align with this socialisation theory and provide important takeaways on learning professionalism in clinical settings, including challenges to learning professionalism and the importance of a tailored strategy befitting a clinical learning environment for teaching professionalism and an explicit professionalism curriculum.

Learning professionalism has always been challenging, and even more so during the pandemic (20). The themes related to the challenges with learning professionalism that emerged from the FGDs consisted of two main subthemes: i) the HC and ii) the COVID-19 pandemic. O’Sullivan (20) defined the HC as ‘the unwritten and unintended lessons acquired by students during their interactions in the learning environment.’ A HC may cause confusion and conflict among students regarding professionalism (20).

This study found that neither students nor teachers focused on professionalism in clinical learning. Clinical learning opportunities enable students to hone various competencies, ranging from clinical and communication skills to professionalism. The amount of subject information that needs to be learned in clinical learning may be a challenge in learning professionalism (21, 22). Moreover, the literature indicates that teaching professionalism has not been explicit (19). This highlights the importance of teaching professionalism explicitly, making it a priority and a focus of the curriculum, as mentioned in the third theme (19, 23). The busy and dynamic healthcare learning environment contributed to students’ workloads, making it harder for them to perform professionally (5, 21). Limited time in clinical learning was also a challenge to learning professionalism (7, 23). Learning culture in the clinical learning environment, as part of the HC, can also hinder learning professionalism (5, 23). Different learning cultures across clinical departments affected the various role models in the clinical learning environment. Role models are one of the strongest influences on students’ professional identity formation, as explained by socialisation theory (7, 19). Negative role models present a challenge to learning professionalism. Unfortunately, this practice is prevalent and unavoidable in clinical learning and may confuse students about professionalism values (19, 24). However, negative and unexpected learning experiences can serve as an opportunity for learning professionalism through debriefing and reflection (19, 23, 24).

Furthermore, the social restrictions caused by the COVID-19 pandemic were barriers to learning professionalism. Some programmes converted their clinical learning fully into online classes to comply with their local government’s COVID-19 restrictions (25). As the situation improved, offline clinical sessions were conducted with additional caution given to the safety of students, faculty members and patients. Thus, offline sessions were limited in time and did not provide a variety of cases, making it even harder for students to interact with patients and learn professionalism (26, 27). The lack of interactions with patients resulted in anxiety and a loss of confidence about student competence (15, 19). This finding highlights the importance of direct patient interactions and the clinical learning environment in learning professionalism (26).

Reflecting on these challenges, the students suggested ideas for learning professionalism that were formulated into strategies tailored to the clinical learning environment, including direct patient interaction, positive role modelling, debriefing and context-specific professionalism.

Direct patient interaction is one of the most important post-pandemic takeaways in learning professionalism. According to socialisation theory, patient interaction through clinical experiences has the greatest effect on student professionalism (7, 19). Various patient interactions enable students to practice their clinical competencies, including professionalism and allow them to observe clinical teachers’ role modelling (19, 20, 23, 28). Students internalise the values learned from role models (e.g. clinical and communication skills, professionalism) through unconscious acquisition and conscious reflection, highlighting the importance of positive role modelling (7, 19). Reflection is key to facilitating individual experiences into meaningful experiences for professional identity formation. It is impossible to fully control and tailor the clinical learning environment to people’s needs (7, 19, 29). Therefore, direct patient interactions and role modelling should be followed by reflection to maximise the impact of these activities on students’ professional identities (7, 19). Students strongly suggested a dedicated time for reflection through debriefing sessions. They preferred observation and feedback about their professionalism and discussions on unexpected clinical experiences, making professionalism a topic explicitly discussed in the curriculum (19, 29). Another strategy suggested in this study is the knowledge teaching of professionalism, which is context-specific or discipline-based. Students must know which aspects of professionalism require attention in each discipline, especially in relation to specific patient characteristics, such as pregnant women, geriatric patients, children, etc. Providing a cognitive base and explicit knowledge for students is part of the strategy for teaching professionalism in medical education (19, 23, 29).

Considering the evidence, this study revealed the need for an explicit professionalism curriculum in clinical learning (15, 19, 23, 29). The literature explained that learning professionalism has not been explicit in medical education, making it harder to prioritise professionalism in teaching, learning and assessment (19, 30). An explicit curriculum should be supported by established standards and expectations for professionalism tailored to the context of the values of an institution’s clinical learning environment. These standards and expectations should be formulated into clear learning objectives that students and teachers can use (19, 20, 23, 29). The students in this study suggested an integrated and longitudinal module for learning professionalism in the clinical setting. This would provide opportunities for dedicated sessions in each clinical rotation for teaching context-specific professionalism and for conducting debriefing sessions after patient interactions. An assessment of professionalism should also be integrated into workplace-based assessments for each clinical rotation (15, 30). By using this explicit curriculum for teaching professionalism in the clinical setting, faculty development efforts are even more important (5, 19, 23). Efforts should focus on having the same perceptions of learning objectives, prioritising professionalism in learning, and teaching context-specific knowledge of professionalism among students, teachers and faculty (19, 23).

Reflecting on the suggested strategies, this study found that teaching professionalism in the clinical environment employs a more teacher-driven strategy on the educational strategies spectrum (31) because of the debriefing sessions, role modelling and knowledge instruction that clinical teachers must provide. This may be the result of Indonesia’s hierarchical and high-power distance culture, which means that change is better accepted when initiated by those with higher power, e.g. clinical teachers (32, 33). However, educational strategy is a spectrum of teacher-driven and student-centred approaches. Therefore, students should always be considered when designing a curriculum and teaching professionalism (15, 30). Another vital takeaway is that learning professionalism should be tailored to the institutional, social and cultural context (23).

Professionalism is conceptualised as an actualisation of humanistic values inside a person. Consequently, professionalism would be better nurtured through practices that evoke humanistic values from within, such as reflective practice and portfolios (5, 6). This study revealed that reflective practice in a hierarchical and high-power distance culture needs to be done in the formally guided debriefing session with the clinical teacher.

The limitation of this study is that it was conducted in a single institution with more female than male respondents. However, the respondent recruitment process—involving students with different numbers of past clinical rotations—was our attempt to reduce any gender biases in the results. Moreover, the results provide important takeaways in designing a professionalism curriculum for the clinical learning environment, as developing a general curriculum to teach professionalism is impossible and should be tailored to each institution’s learning environment (28).

## Conclusion

Learning professionalism came with various challenges, including the COVID-19 pandemic and the HC. Considering these, students highlighted the importance of patient interaction, reflection, role modelling, and an explicit curriculum for learning professionalism. These findings are paramount for curriculum design on teaching humanism and professionalism.

## Figures and Tables

**Table 1:** FGD questions

Questions
**Opening question** What do you think about learning professionalism and humanism in clinical settings?
**Main questions** How did you learn about professionalism and humanism in clinical settings? **Probing**: How was the learning planned? How was the learning implemented? How was the learning assessed? How was the curriculum evaluated? To nurture professional and humanistic physicians, how should the learning be implemented? **Probing**: How should the learning be planned? How should the learning be assessed and evaluated? Why? What are the supporting factors in learning professionalism and humanism? What are the inhibiting factors in learning professionalism and humanism?

**Table 2:** Respondents’ characteristics

FGD code	Respondent	Gender	Year
F1	11 students	4 males	5
		7 females	5
F2	11 students	3 males	5
		8 females	5
F3	9 students	5 males	6
		4 females	6
